# Functional Domains of Androgen Receptor Coactivator p44/Mep50/WDR77and Its Interaction with Smad1

**DOI:** 10.1371/journal.pone.0064663

**Published:** 2013-05-29

**Authors:** Yirong Li, Liantian Tian, Martin Ligr, Garrett Daniels, Yi Peng, Xinyu Wu, Mandeep Singh, Jianjun Wei, Yongzhao Shao, Herbert Lepor, Ruliang Xu, Zhijie Chang, Zhengxin Wang, Peng Lee

**Affiliations:** 1 Department of Pathology, New York University School of Medicine, New York, New York, United States of America; 2 Department of Pathology, Northwestern University, Chicago, Illinois, United States of America; 3 Department of Environmental Medicine, New York University School of Medicine, New York, New York, United States of America; 4 Department of Urology, New York University School of Medicine, New York, New York, United States of America; 5 Department of Biological Sciences and Biotechnology, School of Medicine, Tsinghua University, Beijing, China; 6 Department of Cancer Biology, UT M.D. Anderson Cancer Center, Houston, Texas, United States of America; 7 Cancer Institute, New York University School of Medicine, New York, New York, United States of America; 8 New York Harbor Healthcare System, New York University School of Medicine, New York, New York, United States of America; University of Texas Health Science Center, United States of America

## Abstract

p44/MEP50/WDR77 has been identified as a coactivator of androgen receptor (AR), with distinct growth suppression and promotion function in gender specific endocrine organs and their malignancies. We dissected the functional domains of p44 for protein interaction with transcription factors, transcriptional activation, as well as the functional domains in p44 related to its growth inhibition in prostate cancer. Using a yeast two-hybrid screen, we identified a novel transcription complex AR-p44-Smad1, confirmed for physical interaction by co-immunoprecipitaion and functional interaction with luciferase assays in human prostate cancer cells. Yeast two-hybrid assay revealed that the N-terminal region of p44, instead of the traditional WD40 domain at the C-terminus, mediates the interaction among p44, N-terminus of AR and full length Smad1. Although both N and C terminal domains of p44 are necessary for maximum AR transcriptional activation, the N terminal fragment of p44 alone maintains the basic effect on AR transcriptional activation. Cell proliferation assays with N- and C- terminal deletion mutations indicated that the central portion of p44 is required for nuclear p44 mediated prostate cancer growth inhibition.

## Introduction

Prostate cancer (PCa) is the most common malignancy in men in the United States. In 2011, prostate cancer alone leads to 29% of all newly diagnosed cancers and is the second leading cause of death as a result of cancer [1]. Androgen receptor (AR) is the essential mediator of androgen action and plays an important role in regulating prostate development and differentiation, as well as cancer cell growth and progression [2]. AR is a 110 kDa protein and belongs to the nuclear receptor transcription factor superfamily [3]. Upon activation, AR binds to androgen response elements, interacts with cofactors and transcriptionally activates or represses its target genes [4].

An increasing number of AR cofactors have been identified in recent studies [5], [6], [7]. AR cofactors are divided into coactivators or corepressors. A large number of AR coactivators have been described to promote PCa growth, yet we described several AR coactivators, including p44/Mep50/WDR77, with tumor suppressor function in PCa [8], [9]. Overexpression of Androgen Receptor Trapped clone-27 (ART-27), an AR coactivator, inhibits androgen-mediated cellular proliferation in androgen-dependent cancer cells [10], [11]. Overexpression of AR-associated (ARA) coactivator ARA70α also inhibits LNCaP cell proliferation [8]. p44 has been identified as an AR coactivator and overexpression of nuclear p44 caused growth arrest both in in vitro cell proliferation and in vivo tumor xenografts in AR-dependent manner [9]. Recently, two nuclear export signal (NES) and three nuclear localization signal (NLS) sequences related to its cellular localization have been reported in the p44/WDR77 protein [12].

Several signal transduction pathways, including transforming growth factor-β superfamily (TGF-β) and bone morphogenetic proteins (BMP) pathways, converge with AR signaling and alterations in these pathways have been implicated in PCa development and growth [13]. Smad1 has been identified as a corepressor for AR and inhibits proliferation of androgen dependent prostate cancer cells. Interaction between Smad1 and AR is induced by both BMP and androgen [14].

In this study, we report that Smad1 physically interacts with p44 and the interaction is mediated by AR, subsequently regulating AR transcription activity in the presence of both BMP and androgen. We further delineated three functional domains responsible for AR interaction, AR transcriptional activation and growth inhibition in p44.

## Materials and Methods

### Reagents

Antibodies against AR, Smad1 and β-actin, as well as secondary antibodies, were purchased from Cell Signaling Technology, Inc. (Boston, MA) or Santa Cruz Biotechnology, Inc. (Santa Cruz, CA). Affinity purified p44 antibody was described previously [9].

### Cell Culture and Cell Proliferation Assays

LNCaP cells (From ATCC, a commercial source) and its derivate C4-2B were maintained in RPMI 1640 (GIBCO, Gaithersburg, MD) with 10% heat-inactivated bovine serum (FBS). The androgen-independent LNCaP-AI cells, a derivative of LNCaP, were maintained in RPMI 1640 medium containing 10% charcoal-stripped, heat-inactivated FBS (CSFBS) (Hyclone Laboratories, Inc., Logan, UT) and 5 µg/ml of insulin, as described previously [15]. Cell proliferation was determined by WST-1 as described previously [16]. The assays were done in triplicate.

### Co-immunoprecipitation and Chromatin Immunoprecipitation

LNCaP cells stably expressing NLS-p44 were cultured in 100 mm dish in either androgen (final concentration 10 nM) or androgen free media until 80%–90% confluence. Cells were then transfected with Smad1 plasmid using lipofectamine 2000 (Invitrogen) following manufacturer user manual. 48 hours after transfection, cells were treated with BMP-2 (GenScript) at a final concentration of 100 ng/mL and harvested in lysis buffer (Cell Signaling) supplemented with protease inhibitor cocktail (Sigma). Whole cell lysates were collected by centrifugation at 10,000 rpm for 10 minutes at 4°C and total protein concentration measured using a BCA quantitation assay (Pierce). Lysates were precleared by adding 20 µl resuspended Protein G-Agarose beads (Kirkegaard & Perry Laboratories) 4°C for 30 minutes with rotation. 500 µg total proteins was immunoprecipitated with either 1 µg or 2 µg anti-Smad1 (Millipore) or anti-p44 antisera overnight at 4°C. Antibody was captured with 20 µl of slurry of 50% protein A/G beads equilibrated in lysis buffer by mixing for 4 hours at 4°C, and beads were washed three times with lysis buffer. Bound protein was eluted in 2x loading buffer and analyzed by SDS-PAGE electrophoresis and immunoblotting as described below.

Chromatin IP (ChIP) was performed as described previously [16]. A 145-bp product from −71 to −215 on the promoter of the ß-actin gene and a 215-bp product from −1 to −215 on the promoter of the p21 gene were amplified with primers ActinFR (ActinF: 5-TCCTCCTCTTCCTCAATCTCG-3, ActinR: 5-AAGGCAACTTTCGGAACGG-3) and p21FR (P21F1∶5-GGCTGGCCTGCTGGAACTC-3, P21R1∶5-CAGCTGCTCACACCTCAGC-3), respectively.

### Western Blot Analysis

After electrophoresis on SDS-PAGE, protein was transferred to a nitrocellulose membrane for Western blot analysis. Immunoblots were blocked for 30 min in 3% nonfat dry milk in TBST (20 mM Tris-HCl, pH 7.6, 150 mM NaCl, and 0.1% Tween 20). Blots were incubated with anti-AR, anti-p44 or anti-Smad1 antibodies overnight at 4°C, washed with TBST three times, and incubated for 1.5 h with secondary antibody (1∶2,000) (Amersham Biosciences). Antibodies were diluted with 2% BSA in TBST. The protein bands were detected by an enhanced chemiluminesence kit (Amersham Biosciences).

### Construction of Series of p44 Deletions, Stable Cell Lines and Luciferase Assays

The p44 cDNA was deleted every 200 bp from ether N-terminus or C-terminus to generate series of deletions N8, N6, N4, N2, C8, C6, C4 and C2. These deletions were cloned into plasmid pBabe-NLS and fused with nuclear localization signal (NLS) to give plasmids pBabe-NLS-N8, pBabe-NLS-N6, pBabe-NLS-N4, pBabe-NLS-N2, pBabe-NLS-C8, pBabe-NLS-C6, pBabe-NLS-C4 and pBabe-NLS-C2. Those plasmids were used for luciferase assay or construction of stable cell lines. Construction of stable cell lines and luciferase assay were performed as described previously [9].

### Yeast 2-hybrid Assays

Yeast media were prepared as described previously [17]. Briefly, to screen p44 interacting protein, yeast strain EGY48 (*MATa leu2::6xLexAop-LEU2 trp ura ade his*) was transformed with pSH18-34 reporter vector [URA3, 2 μ, 8xLexAop::lacZ] (Origene Technologies), pLexA or pLexA::p44. Resulting yeast strain was then transfected with a yeast two-hybrid library derived from human prostate (Origene). Transformants were selected and target cDNA was puified as described previously [18]. Amplified DNA was then sequenced and used for further testing.

Direct yeast two-hybrid assays were performed to delineate the structural and functional p44 domains. Yeast strain W303-1A (*MATa leu2-3,112 trp1-1 can1-100 ura3-1 ade2-1 his3-11,15*) [19] was transformed with pSH18-34 reporter vector [URA3, 2µ, 8xLexAop::lacZ] (Origene Technologies) and the appropriate plasmids carrying constructs to be tested [20]. The yeast two-hybrid assays were performed as described previously [20]. In this assay, synthetic androgen R1881 was added to the final concentration of 0.5 µM, or equal volume of solvent (ethanol).

### Statistical Analysis

Yeast two-hybrid, luciferase and cell proliferation assays were all performed in triplicate and repeated three times. Error bars represent standard deviation from triplicates. Statistical analyses from three independent experiments for yeast two-hybrid between androgen and androgen free media, for luciferase assay in different conditions and cell proliferation of different stable cells expressing p44 deletions were performed with pairwise t tests. P-values less than 0.05 were considered statistically significant.

## Results

### Androgen and AR-dependent Interaction between AR Coactivator p44 and Smad1

**Figure 1:**
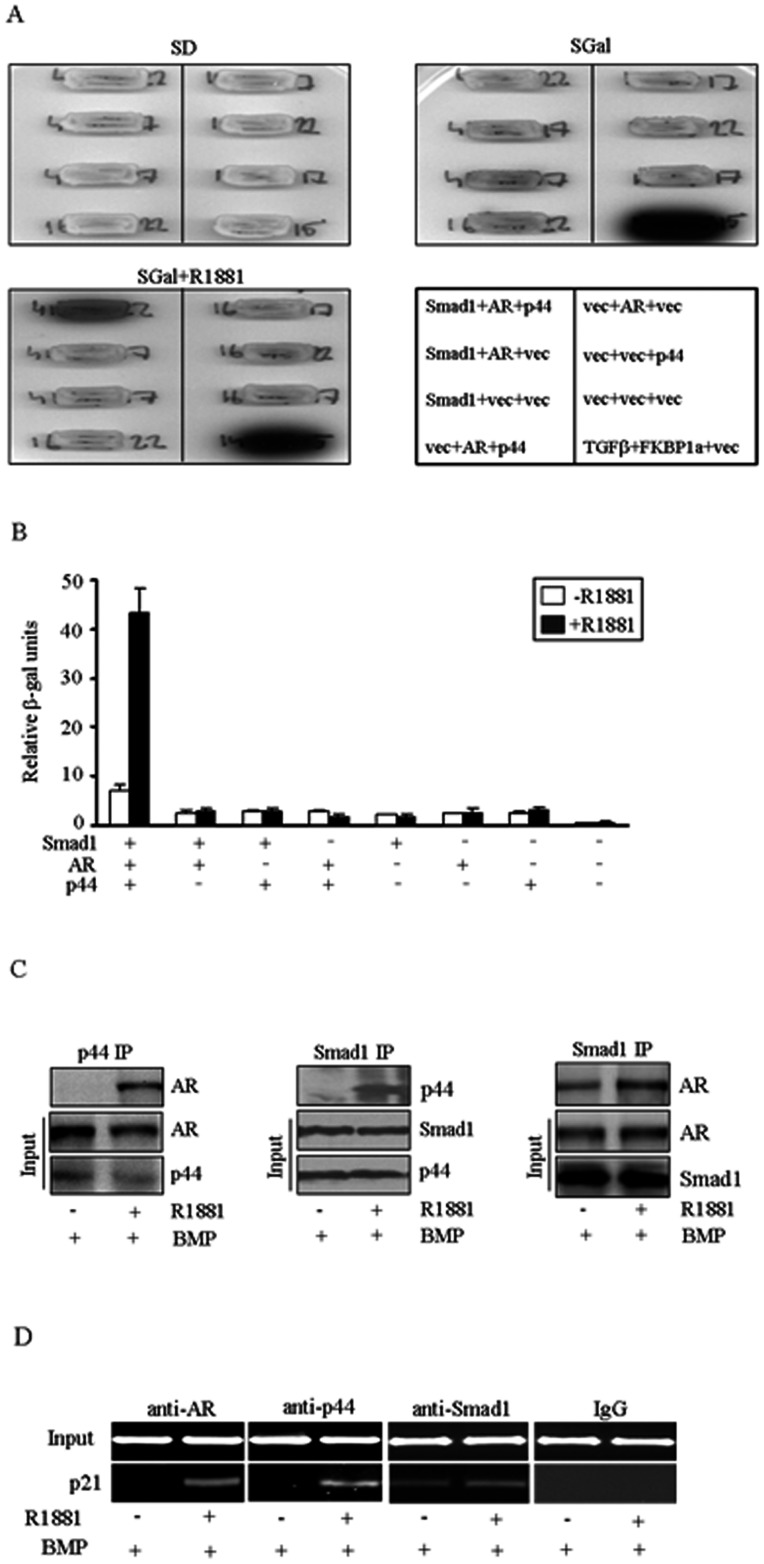
Interaction among AR, Smad1 and p44. (A) and (B) Interaction among AR, Smad1 and p44 in androgen-dependent manner by yeast two-hybrid assay. Different combinations among AR, Smad1 and p44 were tested and p44 interaction with full length Smad1 mediated by AR in androgen-dependent manner. (C) Physical interaction among AR, Smad1 and p44 confirmed by co-immunoprecipitation assay. *Left*, with anti-p44 immunoprecipitation, AR was detectable in the presence of androgen; *Middle*, with anti-Smad1 immunoprecipitation, p44 was in complex with Smad1 in the presence of androgen; *Right*, with anti-Smad1 immunoprecipitation,AR was present in the complex with Smad1 regardless of androgen. (D) ChIP analysis showed that AR, p44 and Smad1 were recruited to the p21 promoter in the presence of androgen and BMP-2.

In order to discover novel proteins interacting with p44, we performed yeast two-hybrid screening, using p44 as bait, against a library derived from mRNA expressed in human prostate. One of the vectors isolated in this screen contained an incomplete open reading frame of Smad1. The expressed protein interacted with p44 in two-hybrid tests (data not shown). We therefore constructed a two-hybrid vector expressing full length Smad1. Full length Smad1, however, did not interact with p44 in two-hybrid tests (Fig. 1A, *right*). We reasoned that p44 and full length Smad1 may interact indirectly, via a mediator. Since both proteins are known to be involved in androgen receptor (AR)-mediated gene regulation, we decided to test whether the introduction of AR into the two-hybrid system will restore p44-Smad1 interaction. Vector only plus TGFβand FKBP1a were used as a positive control. The resulting system then contained p44-LexA fusion that binds to the LexA operators in the reporter plasmid, Smad1 fused to the B42 transcription activation domain, and AR. Coexpression of Smad1, p44, and AR resulted in a positive two-hybrid signal (Fig. 1A, B), suggesting that AR binds both to p44-LexA (which binds to the LexA operator on the DNA), and Smad1-B42, recruiting the transcription activation domain to the promoter and thus activating reporter gene expression. This phenomenon was observed only in the presence of the synthetic androgen R1881 in the media, confirming that the interaction of p44 and Smad1 is AR-dependent.

To perform a more careful examination of the interactions among p44, AR and Smad1 in mammalian cells, serials of co-immunoprecipitation were performed using LNCaP cells stably expressing NLS-p44. With anti-p44 antibody, we were able to precipitate and then detect AR in androgen supplemented media but not in androgen-free media (Fig.1C, left panel), which is consistent with previous studies [9]. Similarly, p44 was precipitated using anti-Smad1 antibody in cells grown under the treatment of both BMP-2 and androgen, but not in BMP-2 and androgen free condition (Fig. 1C, middle panel). This data suggests that interaction between Smad1 and p44 requires the presence of both androgen and BMP-2. Interestingly, when the immuno-complex was precipitated by anti-Smad1 antibody and probed with anti-AR antibody, AR was detected in immuno-complex extracted from cells grown in both media supplemented with androgen (final concentration of 10 nM) and in androgen free media (Fig.1C, right panel). Our data suggest that treatment with either androgen or BMP-2 can cause the interaction between Smad1 and AR, ie the activation of either AR or Smad1 signaling pathway triggers the formation of this complex, indicating that Smad1 plays an essential role in AR function. To further confirm that AR-p44-Smad1 is in an endogenous complex, we examined their interactions only at endogenous levels by co-IP in C4-2B prostate cancer cells. The result confirmed that AR, p44 and Smad1 form an endogenous complex (Fig. S1). Furthermore, we examined the recruitment of the AR-p44-Smad1 complex to the promoter of p21,a well-characterized AR target gene, by ChIP in C4-2B cells. AR, p44 and Smad1 were recruited to the p21 promoter in the presence of androgen and BMP-2 (Fig. 1D), confirming that AR, p44 and Smad1 form a transcription complex and co-regulate target genes.

### Structural Domains Responsible for Transcriptional Activation and Interaction in p44

**Figure 2:**
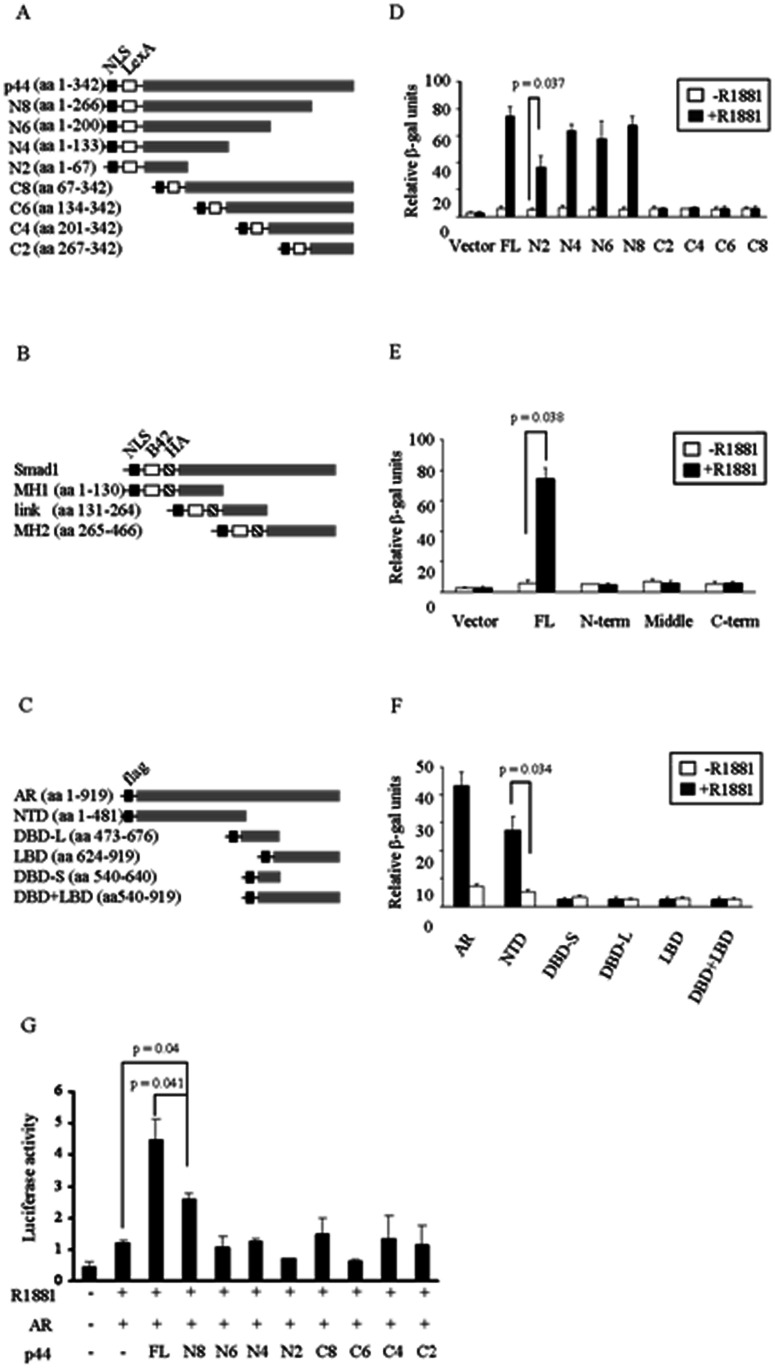
Identification of domains for interaction among AR, p44 and Smad1. (A), (B) and (C) Schematics of construction of yeast two-hybrid plasmids for p44, Smad1 and AR. (D) The extreme N-terminus (N2, p44, AA1-70) was able to sustain reporter expression in yeast two-hybrid reporter assay (p = 0.017). (E) Only the full length Smad1 is able to interact with AR and p44 (p = 0.038). (F) The NTD domain of AR was able to drive the expression of the reporter gene in the presence of androgen with full length Smad1 and p44 (p = 0.034). (G) Luciferase assay also revealed that N-terminus (N8, p44 AA1-266) mediates maximal AR transcriptional activation (p = 0.041).

To determine the p44 domain(s) that are involved in the tripartite interaction, we constructed two-hybrid vectors containing individual domains or fragments of AR, Smad1, and p44. The domain structure of p44 is unknown. Therefore we created a series of N- and C-terminal 200 bp deletion mutants (Fig. 2A). Smad1 domains were divided into the MH1 and MH2 domains, and the intervening linker domain (Fig. 2B). Domains in AR were divided into the N-terminal domain (NTD), DNA-binding domain (DBD), and ligand-binding domain (LBD) (Fig. 2C). First, we tested the interactions between full length Smad1 and different fragments of p44. Only the fragments containing the extreme N-terminus of p44 were able to sustain reporter expression (Fig. 2D). The shortest N-terminal fragment displayed a somewhat lower level of reporter induction, therefore it appears that the sequence critical for the interaction is located around 70 aa (N2, p = 0.037) from the N-terminus of p44 (Fig. 2D). Next, we tested the interaction among individual domains of Smad1, full length AR and p44. None of the individual domains were able to activate reporter expression on their own. Only the full length Smad1 interacted with AR and p44 (p = 0.038) (Fig. 2E). When we tested individual domains of AR in the presence of full length Smad1 and p44, only the NTD domain was able to drive the expression of the reporter gene (p = 0.034) (Fig. 2F).

In order to identify the domain of p44 responsible for AR mediated transcriptional activation, the same serial deletions of p44 were used for transfection in PC3 cells and their effects were examined using dual luciferase assay with a 4XARE luciferase reporter. Consistent with its role as AR coactivator, full length p44 significantly increased levels of AR mediated transcriptional activation (Fig. 2G). On the other hand, serial deletions of p44 displayed varied effects. Deletion mutant N8 displayed 75% of the androgen receptor mediated transcriptional activation observed with full length p44 (p = 0.041) (Fig. 2G). Other deletion mutants showed little to no transcriptional activation (Fig. 2G).

### Smad1 and p44 on AR-mediated Transcription Activation

**Figure 3:**
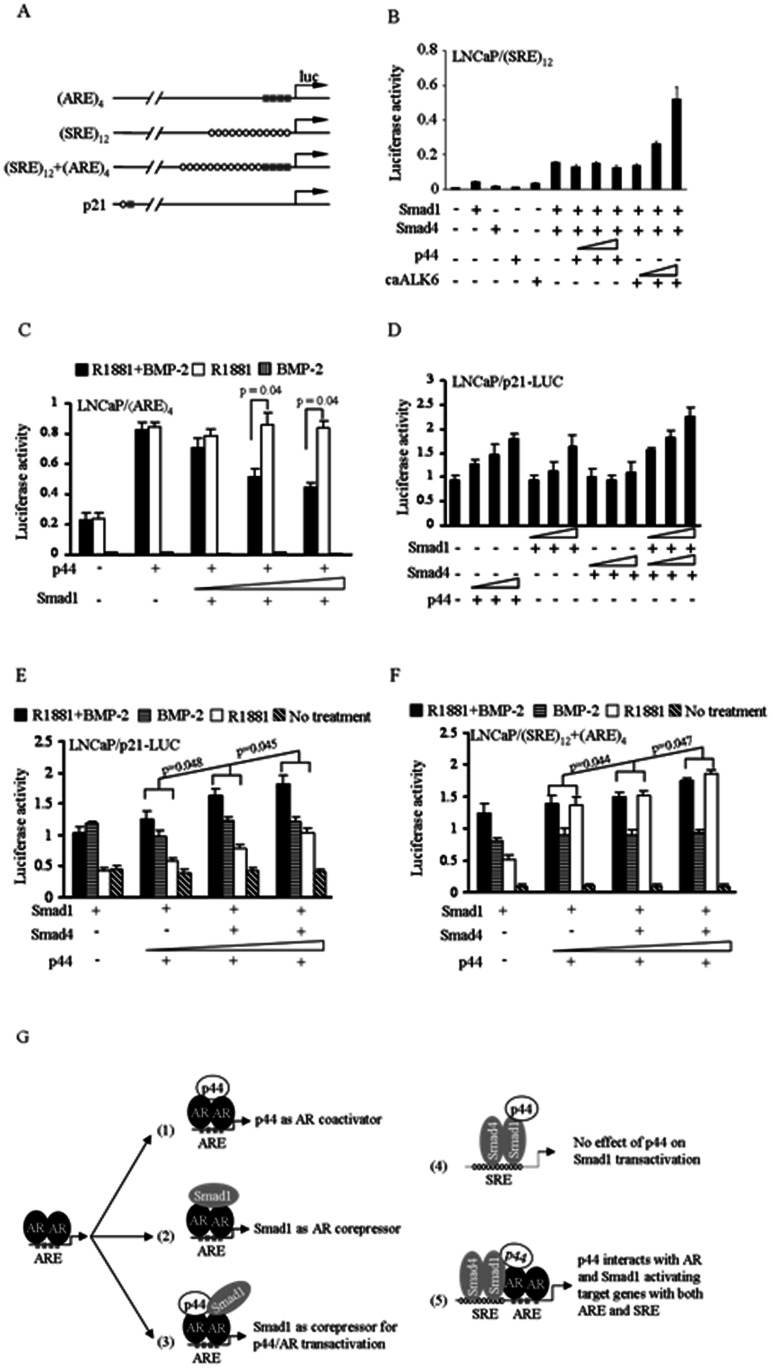
Smad1 and p44 on AR-mediated transcription activation. (**A**) Schematics of construction of luciferase reporters with androgen response element (ARE) or Smad1 response element (SRE). (B) Individual Smad1, Smad4 and p44 only showed basal level of transcription activation from the synthetic Smad reporter in the presence of BMP-2. Co-transfection of Smad1 and Smad4 dramatically increases the Smad-dependent transcription activation increase. p44 did not have any effect on Smad1/Smad4-dependent activation of the reporter. (C) Smad1 acts as a corepressor on p44-dependent stimulation of AR-dependent transcription activation (p = 0.04 in luciferase assay). (D) Both p44 and Smad1 increase transcription activation of the reporter with p21 promoter in the presence of both R1881 and BMP-2. (E) and (F) The interplay of p44 and Smad1/Smad4 on transcription activation with reporter containing p21 promoter and reporter containing synthetic ARE and SRE. Smad1 alone had an inhibitory effect on p44-activated transcription at low doses of p44 and this inhibitory effect disappeared in the presence of Smad4 (p<0.05). (G) Models of interplay among AR, p44 and Smad1 on transcription activation.

Smad1 acts as a corepressor [14] but p44 serves as a coactivator of AR activity [21]. To determine whether p44 is a Smad1 coactivator or Smad1 influences p44 coactivation of AR, we constructed a series of luciferase reporters: reporter with 4X ARE (androgen response element: AGAACAGCAAGTGCT) [22], reporter with 12X SRE (Smad1 response element: CAGACA), reporter with 12X SRE and 4X ARE, reporter containing p21 promoter (Fig. 3A).

First we tested whether p44 is a cofactor of Smad1/Smad4 complex acting independently of AR with the synthetic Smad1 reporter containing 12X SRE (Fig. 3B). As shown in Fig. 3B, Smad1, Smad4 or p44 alone only showed basal level of transcription activation from the synthetic Smad reporter in the presence of BMP-2. When Smad1 and Smad4 were transfected together, the Smad-dependent transcription activation increased ten fold. Addition of increasing amounts of p44 did not have any effect on Smad1/Smad4-dependent activation of the reporter. As expected as positive control, transfection of the constitutively active BMP type 1 receptor caALK6 led to increased Smad1/Smad4-dependent transcription, confirming that BMP/Smad pathway was intact in the LNCaP cells.

Next, we asked whether Smad1 exerts an effect on p44-dependent stimulation of AR-dependent transcription activation in AR-expressing LNCaP cells. Luciferase assay showed that p44 increased transcription activation approximately 3 fold from the reporter under the control of 4XARE synthetic promoter in the presence of R1881 (Fig. 3C). Co-transfection with Smad1 in the presence of both R1881 and BMP-2 led to dose-dependent inhibition of AR-reporter transcription (p = 0.04 and p = 0.04), suggesting that Smad1 acts as a corepressor on p44-dependent stimulation of AR-dependent transcription activation, consistent with published data [23]. The promoter of the cyclin-dependent kinase inhibitor p21 contains one ARE and one binding site for Smad1. Both p44 and Smad1 are able to increase transcription activation in dosage manner with reporter containing p21 promoter in the presence of both R1881 and BMP-2 (Fig. 3D). Individual Smad4 didn’t show increase on transcription activation while cotransfection of Smad1 and Smad4 synergistically increased transcription activation (Fig. 3D).

Finally, to study the interplay of p44 and Smad1/Smad4 on transcriptional activation, we tested various combinations of p44 and Smad1/Smad4 with synthetic reporter (12XSRE and 4XARE) or reporter containing p21 promoter (native single copy of ARE and SRE) in the presence and absence of their respective ligands. As shown in Fig. 3E, without any ligand, the reporter maintains basal transcriptional activation. In the presence of R1881, p44 increased transcription activation in dose-dependent manner (Fig. 3E, white bar, p = 0.048 and 0.045). In contrast to Smad1 as AR corepressor with ARE promoter in the presence with androgen, Smad1 increased trasnscriptional activation with in the presence of BMP with promoter with dual ARE and SRE. Interestingly, p44 and Smad1 have additive, not synergistic effects in transcriptional activation (Fig. 3E, black bar) with dual ARE and SRE promoter in the presence of both R1881 and BMP-2, suggesting a dominant role of p44 in AR-p44-Smad1 complexes on transcription activation. Similar results were obtained using reporter with synthetic ARE and SRE (Fig. 3F, p = 0.044 and 0.047).

### Domains Responsible for Inhibition of Growth

**Figure 4:**
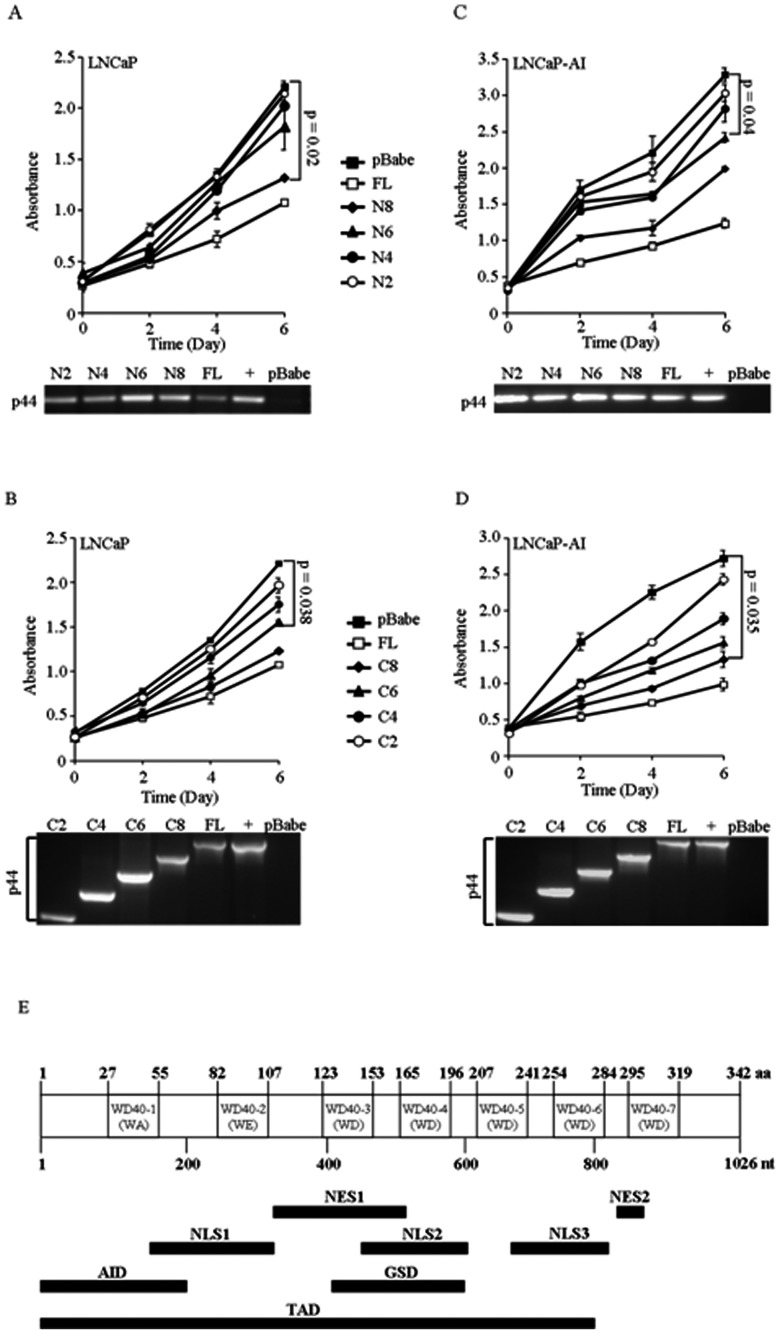
Characterization of domains responsible for inhibition of growth. (A) Upper panel: Proliferation of cells stably expressing truncated N-terminal p44 in LNCaP cells. Truncated NLS-N8 showed significantly inhibitory effects on cell proliferation in LNCaP cells (p = 0.02). Lower panel: RT-PCR for expression of truncated constructs. (B) Upper panel: Proliferation of cells stably expressing truncated C-terminal p44 in LNCaP cells. Truncated NLS-C8 and NLS-C6 (p<0.05) displayed inhibitory effects on cell proliferation in LNCaP cells. Lower panel: RT-PCR for expression of truncated constructs. (C) Upper panel: Proliferation of cells stably expressing truncated N-terminal p44 in LNCaP-AI cells. Truncated NLS-N8 and NLS-N6 (p = 0.04) showed significant inhibitory effects on cell proliferation in LNCaP-AI cells. Lower panel: RT-PCR for expression of truncated constructs. (D) Upper panel: Proliferation of cells stably expressing truncated C-terminal p44 in LNCaP-AI cells. Truncated NLS-C8 and NLS-C6 (p = 0.035) displayed significant inhibitory effects on cell proliferation in LNCaP cells. Lower panel: RT-PCR for expression of truncated constructs. (E) Schematics of structure of p44. WD40-1 and WD40-2 are atypical WD40 repeats. NLS1 is dominant while NLS2 and NLS3 are atypical NLS. NES1 is dominant but NES2 is atypical NES. AID: AR Interaction Domain. TAD: Transcriptional Activation Domain. GSD: Growth Suppression Domain.

Nuclear expression of p44 is decreased in PCa and overexpression of nuclear p44 caused growth arrest both in *in vitro* cell proliferation and *in vivo* tumor xenografts in an AR-dependent manner [9]. To identify domain(s) responsible for the function of growth inhibition in p44, we constructed a series of stable cell lines expressing nuclear localization signal (NLS) fusion with truncated p44 in both LNCaP cells (androgen-dependent prostate cancer cell line) and LNCaP-AI cells (an androgen-independent derivative of LNCaP cells) with at least three clonal lines for each deletion mutation. WST-1 assay indicated that NLS-p44 showed significant inhibitory effects on cell proliferation in LNCaP and LNCaP-AI cells (Fig. 4A and C). There is a gradual decrease in the growth inhibition ability for increasing deletions from either N-terminus or C-terminus. Deletion mutants NLS-N8 and NLS-C8 showed significant inhibitory effects on cell proliferation in LNCaP and LNCaP-AI cells (p = 0.02 and p = 0.035) (Fig. 4A and D). Truncated NLS-N4 and NLS-C4 displayed substantially diminished growth inhibitory effects in LNCaP and LNCaP-AI cells (Fig. 4A and D). Specifically, truncated NLS-N4, NLS-N2, NLS-C4 and NLS-C2 showed minimal growth inhibitory effects, suggesting that the fragment overlapping between N6 and C6 is critical for p44 growth inhibitory effects (Fig. 4B, p = 0.038 and Fig. 4C, p = 0.04) on prostate cancer cell proliferation.

## Discussion

In this study, we determined the functional domains of p44 responsible for protein-protein interaction with AR and Smad1 transcription factors, transcriptional activation, as well as the functional domains related to growth inhibition in prostate cancer. Using a yeast two-hybrid screen and co-immunoprecipitation, we have identified a novel transcription complex AR-p44-Smad1 in prostate cancer cells. The N-terminus of p44 was found to be not only responsible for its interaction with AR and Smad1, but also for its AR transactivation. The central portion of p44 is revealed to be required for nuclear p44 mediated prostate cancer growth inhibition in cell proliferation assays with series of deletion mutations (Fig. 4E).

p44 was initially identified as a subunit of the methylosome complex and later characterized as a steroid receptor coactivator that enhances androgen receptor (Fig. 3G-1) as well as estrogen receptor mediated transcriptional activity in a ligand-dependent manner. Although it is established that p44 acts as a coactivator of AR, the components of AR transcription complexes are still elusive. Of note, p44 functions as an AR coactivator in cell based luciferase assays, while it inhibits AR transcriptional activation in cell free in vitro transcriptional assays [24], indicating that additional factor(s) are involved for p44 coactivator ability. To discover novel proteins in complex with AR and p44, we employed a yeast two-hybrid screen using p44 as bait, and found Smad1 to interact with p44. This is consistent with a previous report of Smad1 and AR interaction [14]. Co-IP experiments also confirmed that AR, p44 and Smad1 physically interact in complexes. Different domains from AR, p44 and Smad1 are responsible for the tripartite interaction with two-hybrid assay. The N-terminus of p44 and NTD of AR mediates the interaction with full length Smad1. It is important to note the tripartite nature of the interaction between AR, p44 and Smad1.

Five WD40 repeats have been identified in the C-terminus region of p44 and these WD40 repeats are speculated to mediate the protein-protein interactions in p44 multiple protein complexes. Recently, Antonysamy et al. determined the crystal structure of human PRMT5 in complex with MEP50 (methylosome protein 50), bound to an S-adenosylmethionine analog and a peptide substrate derived from histone H4. The interaction between seven-bladed β-propeller MEP50 and the N-terminal domain of PRMT5 mediates the complexes of PRMT5 and MEP50 [25]. However, our data showed that only the N-terminal fragment (approximately 70 aa from the N-terminus) in p44 is necessary for its interaction with AR and Smad1. Detailed analysis revealed two atypical WD repeats resides in the N-terminus with WA and WE instead of WD sequence (Fig. 4E). In terms of functional domains of AR, our data shows that the N-terminal domain (NTD) of AR was responsible for the tripartite interaction with p44 and Smad1 in an androgen-dependent manner. Unlike AR and p44, only full length Smad1 was able to mediate the tripartite interaction.

In study of the domain(s) responsible for AR transcriptional activation, we found both N-terminus and the central region are critical for p44 to maintain maximum effect on AR transcription activation, while N-terminus (up to N6) alone exhibits minimal effect on AR transcription activation. Deletion N8, containing the 266 amino acids from N-terminus is the minimal length for exerting effect on AR transcriptional activation (Fig. 4E). Interestingly, p44 acts as coactivator for AR while Smad1 serves as an AR transcriptional corepressor [14], [21] (Fig. 3G-1, 2). We also addressed the question if p44 functions as cofactor for Smad1/4 and found no effect of p44 on Smad transcriptional activation (Fig. 3B and 3G-4). Another intriguing question is that whether Smad1 has additive or synergistic effects with p44/AR transcriptional activation. Our data indicates that Smad1 acts as a corepressor for AR/p44 (Fig. 3C and 3G-3), consistent with published data by Qiu et al that Smad1 is an AR corepressor [14]. With p21 promoter, there is a p44 dosage-dependent increase in the level of transcription in the presence of R1881, but not BMP (Fig. 3E, white bar). It seems that there is additive, but not synergistic, effect for Smad1 with p44 in increasing the levels of transcription further (Fig. 3E, black bar) in the presence of both androgen and BMP. However, considering Smad1 functions as a corepressor for AR/p44 (Fig. 3C), there is also a reversion of transcriptional inhibition from ARE (Fig. 3C, 3G-3) to activation for dual responsive elements (ARE and SRE) in the presence of both androgen and BMP (Fig. 3E, F, G-5).

Nuclear p44 inhibits PCa cell proliferation [9]. We created a series of plasmids with nuclear localization signal (NLS) fused with truncated p44. Of all the mutant constructs of NLSp44 we tested, the strongest growth inhibition occurred using the NLS-N8 deletion mutant but NLS-C8 and NLS-C6 also displayed inhibition on cancer cell growth. Based on this data, we reasoned that the fragment overlapping between N6 and C6 is critical for inhibitory effects on cell proliferation. Our previous data suggested that NLS-p44 growth inhibition is AR-dependent. In this study, we demonstrated that N-terminal fragment (around 70 amino acids from the N-terminus) in p44 is critical for its interaction with AR and Smad1. NLS-C8, NLS-C6 deletions do not contain this N-terminal fragment (around 70 amino acids from the N-terminus), but they still exhibit repression on prostate cancer cell growth. We propose there may be other unknown factor(s) mediating inhibition of NLS-C8 and NLS-C6 through AR interaction, while the fragment between 123 and 224 amino acids (area overlapping between N6 and C6) maybe essential for growth inhibition in prostate cancer cells.

Worthy to mention, the p44 functions are regulated by its subcellular localization [9]. It functions in the nucleus as a nuclear receptor cofactor to inhibit cell proliferation and promote cell differentiation. In contrast, it is required for cell proliferation in the cytoplasm. There are several nuclear localization and nuclear expert signals (NLS and NES), NLS1-3 and NES 1-2, identified and the predominant NLS is non-conventional (Fig. 4E) [12], [26]. Part of NLS1 is inside of N-terminus of p44 mediating the interaction with Smad1 (Fig. 4E). The transactivation domain in p44 contains an intact NLS1, NLS2, NES1 and NES3 (Fig. 4E). NLS2 locates inside the domain responsible for growth inhibition. These signaling peptides may be important for the function of domains. Whether and how Smad1 regulates the p44 functions in the cytoplasm versus in the nucleus needs further investigation.

In summary, we have identified AR-p44-Smad1 transcription complexes. This result provides better understanding on the molecular mechanisms in regulation of AR transcriptional activation. A balance is present between Smad1-mediated negative regulation and p44-mediated positive regulation of AR activity. Of interest would be the dominant role of p44 in AR-p44-Smad1 complexes on transcriptional activation. We also identified three different regions related to interaction with AR, AR transcriptional activation, and prostate cancer growth inhibition in p44. Our studies provide new insights into the correlation between structures and functions of p44.

## Supporting Information

Figure S1 **The interaction among AR, p44, Smad1 was detected by Co-immunoprecipitation in C4-2B cells.** AR-p44-Smad1 is in an endogenous complex.
(PPT)
