# Supplementary figures and images for: Functional Domains of Androgen Receptor Coactivator p44/Mep50/WDR77and Its Interaction with Smad1
Source: PLoS One. 2013 May 29;8(5):e64663. doi: 10.1371/journal.pone.0064663 (PMC3667176; doi:10.1371/journal.pone.0064663)

## Slide 1
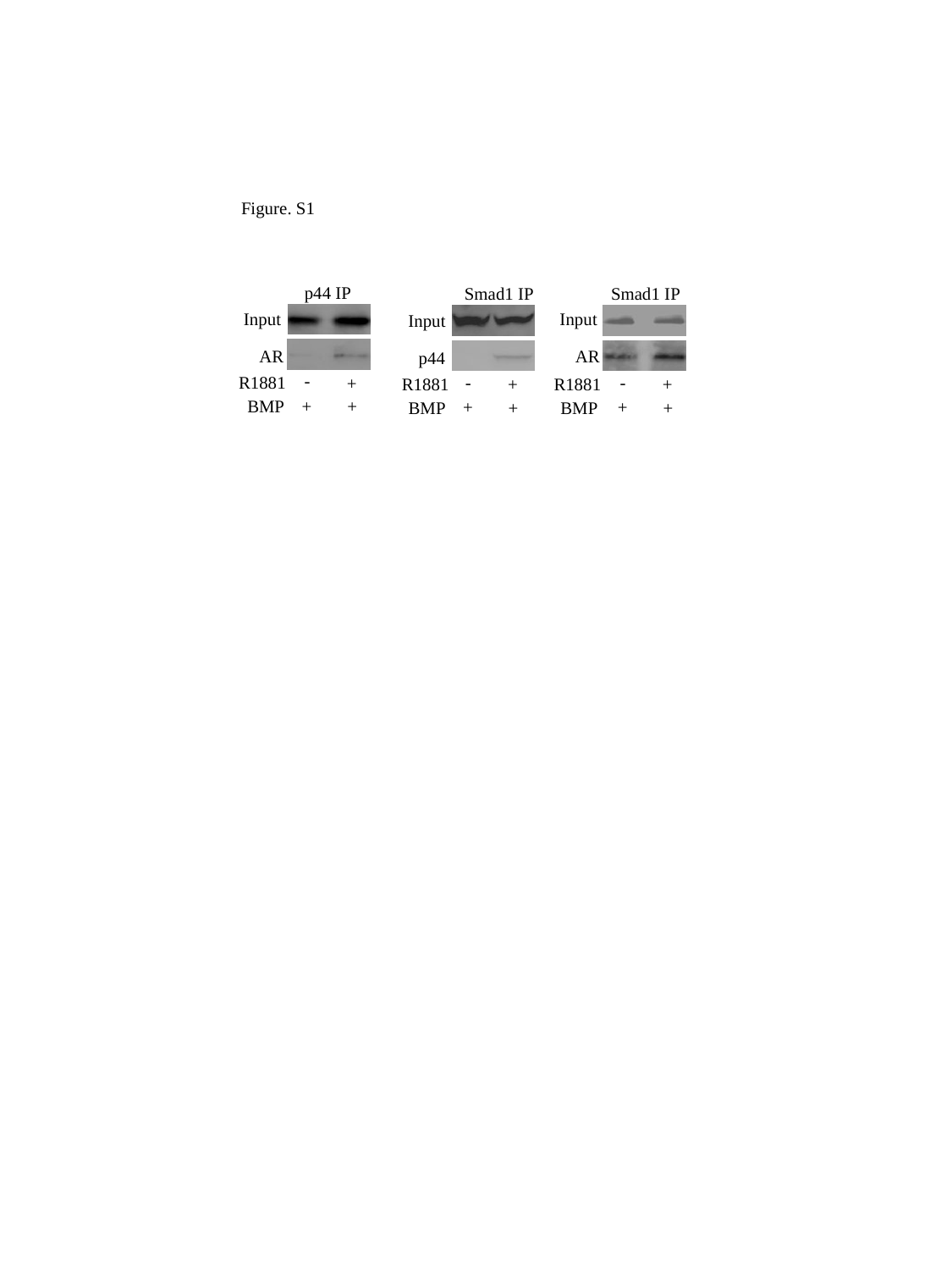

Figure. S1
p44 IP
Smad1 IP
Smad1 IP
Input
Input
Input
AR
AR
p44
-
-
-
R1881
+
R1881
R1881
+
+
+
+
BMP
+
+
+
+
BMP
BMP

Supplement: Figure S1 — The interaction among AR, p44, Smad1 was detected by Co-immunoprecipitation in C4-2B cells. AR-p44-Smad1 is in an endogenous complex. (PPT) [file pone.0064663.s001.ppt]
